# Genomic and Metabolomic Comparisons Provide New Insights into Plant Cell Wall Degradation, Mating Diversity and Secondary Metabolites in Brown and White Commercial *Hypsizygus marmoreus* Varieties

**DOI:** 10.3390/ijms27125372

**Published:** 2026-06-14

**Authors:** Chenli Zhou, Wenyun Li, Yan Li, Ting Guo, Junjun Shang, Lihua Tang, Wenjun Mao, Jianing Wan, Dapeng Bao, Yingying Wu, Ruiheng Yang

**Affiliations:** Key Laboratory of Edible Fungal Resources and Utilization (South), National Engineering Research Center of Edible Fungi, Key Laboratory of Agricultural Genetics and Breeding of Shanghai, Institute of Edible Fungi, Shanghai Academy of Agricultural Sciences, Shanghai 201403, China; zhouchenli@saas.sh.cn (C.Z.); liwenyun1209@126.com (W.L.); liyan03@saas.sh.cn (Y.L.); guoting2018@hotmail.com (T.G.); shangjunjun@saas.sh.cn (J.S.); lhtang2007@163.com (L.T.); mwjtry123@126.com (W.M.); wanjianing@163.com (J.W.); baodapeng@saas.sh.cn (D.B.)

**Keywords:** genomics, metabolomics, carbohydrate-active enzymes, mating-type locus, melanin

## Abstract

*Hypsizygus marmoreus* (Peck) H.E. Bigelow is a commercial edible mushroom includes two primary commercial varieties: brown and white. To reveal the genetic and metabolic differences between these two varieties, genomic and metabolomic comparisons of the white strain F4 and the brown strain B5-15 were performed. The assembled genome sizes were 40,851,948 bp for F4 and 41,902,673 bp for B5-15. Molecular clock analysis estimated that *H. marmoreus* diverged from *Termitomyces* sp. approximately 59.4 million years ago during the Paleocene based on the genomic information. The two genomes showed little difference in the gene compositions related to β-Glucosidase and certain lignin degrading auxiliary enzymes. In contrast, the structures of the mating-type loci, including gene copy numbers and the transcriptional orientation of open reading frames, differed between the varieties, and it exhibited higher mating-type locus diversity. Comparative genomic analysis further indicated that the brown strain can biosynthesize melanin-like compounds using chorismate as the starting molecule, with tyrosinase acting as a key enzyme. Moreover, metabolomic profiling based on principal component analysis (PCA) and orthogonal partial least squares-discriminant analysis (OPLS-DA) revealed distinct metabolic profiles between the two varieties. Collectively, these findings improve our understanding of the genetic basis underlying the phenotypic differences between the two *H. marmoreus* varieties.

## 1. Introduction

*Hypsizygus marmoreus* (Peck) H. E. Bigelow, which belongs to Agaricales, is a commercial edible mushroom [1]. Due to its high nutritional and medicinal value, this mushroom is very popular and widely accepted across East Asian regions, including China and Japan [2,3,4]. It is considered a promising food for improving human health and preventing diseases. To date, two main varieties of this mushroom are widely cultivated for commercial production: the white type and the brown/grey type [5]. These two cultivars exhibit distinct differences in phenotypic characteristics, such as color, taste, and other traits. Currently, multiple omics studies have been reported to explore secondary metabolic pathways, stress responses, developmental mechanisms and other key biological processes in *H. marmoreus* [6,7,8,9,10]. However, there have been few genomic studies elaborating the genetic differences between white and brown *H. marmoreus*.

As a white-rot fungus, *H. marmoreus* can secrete carbohydrate-degrading enzymes to degrade lignocellulose for absorbing growth substances [11,12,13,14,15]. The expression of these lignocellulolytic enzymes is associated with cultivated properties and yields in edible and medicinal fungi [13,15,16]. Therefore, a deeper understanding of the genetic basis of lignocellulose degradation in mushrooms may facilitate the development of more effective strategies for selecting and utilizing feedstocks, thereby improving the efficiency of cultivation. Genomics is considered a powerful approach for studying the mechanisms of plant cell wall degradation, and it has been widely used to uncover lignocellulose degradation in mushrooms [17,18,19]. Several studies have indicated that differences in lignocellulolytic enzymes exist not only among different strains of the same species, but also among different monokaryons derived from the same strain [20,21]. Beyond differences in agronomic traits, the white and grey strains also show distinct cultivation formulations [1], which may reveal the genetic differences presenting in the two varieties. However, no comparison of lignocellulolytic enzyme genes has been conducted between the two varieties.

*H. marmoreus* is known as a tetrapolar heterothallic basidiomycete [22,23]. In this mating system, genes in the A locus encode a homeodomain transcription factor, while those at *B* locus encode both peptide pheromones and receptors [22]. Mating-type genes control sexual development and serve as the key genetic foundation for strain differentiation and cross-breeding [11,24]. In addition, mating frequency and allelic frequencies are associated with geographical distribution [22,25]. Given the importance, mating systems have been characterized in several mushroom species in terms of genetic structure and function, including *Agaricus bisporus* (J.E. Lange) Pilát [26,27], *Flammulina filiformis* Z.W. Ge, X.B. Liu & Zhu L. Yang [25,28,29], *Lentinula edodes* (Berk.) Pegler [30,31,32], *Pleurotus djamor* (Rumph. ex Fr.) Boedijn [33], and *Volvariella volvacea* (Bull.) Singer [34]. All these results revealed the presence of high genetic diversity among different strains. Up to now, the genetic structure and evolutionary diversity of mating-type (*MAT*) genes in *H. marmoreus* has been studied, which also revealed the gene contents were variable [35]. However, the detailed differences in mating-type genes between white and brown strains remain poorly understood, and clarifying these distinctions is critical for the accurate identification and differentiation of the two varieties.

The most significant phenotypic difference between the two varieties is surface color: one is brown (or gray), while the other is white. Brown or gray pigments have been identified as melanin in various mushrooms, e.g., *A. bisporus* [36], *Auricularia auricula* (L.) Underw [37] and *L. edodes* [38]. Melanin synthesis is also involved in pigmentation of brown *H. marmoreus* [39,40]. Two melanin synthesis pathways have been reported in fungi, the 8-dihydroxynaphthalene (DHN) pathway and the L-3,4-dihydroxyphenylalanine (L-DOPA) pathway, of which the L-DOPA pathway is the more common route for melanin production [41]. In this pathway, tyrosinase is the most important enzyme, and it has also been identified in *A. bisporus* [36], *A. auricular* [42] and *Grifola frondosa* (Dicks.) Gray [43]. Based on the study from bulked segregant analysis (BSA) and comparative transcriptome analysis, a cytochrome P450 was regarded as the candidate causal gene for the melanogenesis in *H. marmoreus* [39,40]. The associated genes showed no direct correlation with tyrosinase. The genomic comparison between the two varieties may provide other ways to further elucidate the genetic background and tyrosinase functions.

In this study, genomic and metabolomic comparisons between two varieties of *H. marmoreus*, white and brown, were presented, and this information might help to elucidate the mechanisms of plant cell wall degradation, mating diversity and melanin biosynthesis.

## 2. Results

### 2.1. Genome Sequencing and General Features

For strain F4, a total of 150,086 polymerase reads (2,860,968,946 bp) were generated from the PacBio platform, with an average read length of 19,062 bp, providing approximately 88.2-fold coverage of this genome. After filtering the low-quality reads, 258,199 subreads with an average length of 5743 bp were retained. After filtering the low-quality reads, 67,421,744 high-quality Illumina reads (9,955,446,419 bp) were obtained. Finally, an assembly of 40,851,948 bp with 49.4% GC content across 98 scaffolds was obtained. A total of 12,411 putative genes were predicted (Table 1), 4434 (58.85%) of which encoded proteins with homologous sequences in the Swiss-Prot protein databases (Table 1). The numbers of 5.8S rDNAs, 18S rDNAs, 28S rDNAs and tRNAs identified were 4, 4, 6 and 331, respectively.

Compared to strain F4, the genome size of B5-15 (41,902,673 bp) was larger than that of F4. And a higher number of predicted genes (12,618) was also detected in B5-15. However, only 301 genes coding tRNA were identified in B5-15, which was lower than that found in F4 (331). The most notable differences between the two strains were the copy numbers of 5S, 5.8S, 18S and 28S rDNA genes. For each of these rRNA genes, the copy number in B5-15 was 20 times higher than that in F4.

The two genome assemblies achieved BUSCO completeness scores of 95.86% and 96.93%, indicating high accuracy and near-complete representation of gene sets. Homologous genes (e-value < e^−10^) between the two strains were detected using BLAST (https://blast.ncbi.nlm.nih.gov/, accessed on 30 March 2026), revealing 242 unique genes in strain F4 and 286 unique genes in B5-15 (Figure S1). Synteny block analysis showed that 20,400 collinear genes (25,029 of total genes) were collinear and shared between the two strains, accounting for 81.51% of the total genes.

### 2.2. KOG and KEGG Annotations

**Figure 1:**
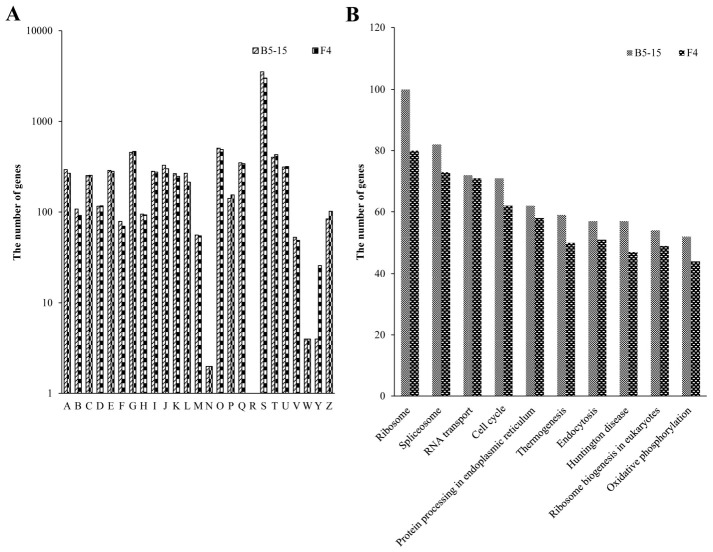
KOG and KEGG annotations of B5-15 and F4. (**A**) KOG; (**B**) KEGG.

A total of 4768 and 4674 genes were assigned into known functional categories based on the KOG database for B5-15 and F4, respectively (Figure 1A, Table S1). The most enriched genes involved in the biological functions were similar in both strains. However, the predicted genes of carbohydrate transport and metabolism (469), signal transduction mechanisms (432), intracellular trafficking, secretion, and vesicular transport (320), energy production and conversion (256), inorganic ion transport and metabolism (154), cytoskeleton (102) and nuclear structure (26) were more abundant in strain F4 than in strain B5-15 (458, 404, 315, 254, 141, 84 and 4, respectively). In contrast, the numbers of other genes were higher in strain B5-15.

A total of 5365 in strain B5-15 and 4795 genes in strain F4 were annotated based on KEGG-predicted pathways (Table S2). In both strains, the most enriched pathways were Ribosome, spliceosome, RNA transport, cell cycle and protein processing in the endoplasmic reticulum (Figure 1B). The number of genes involved in the top five pathways was higher in B5-15 than in strain F4 (Figure 1B).

### 2.3. Phylogenomic Analysis

**Figure 2:**
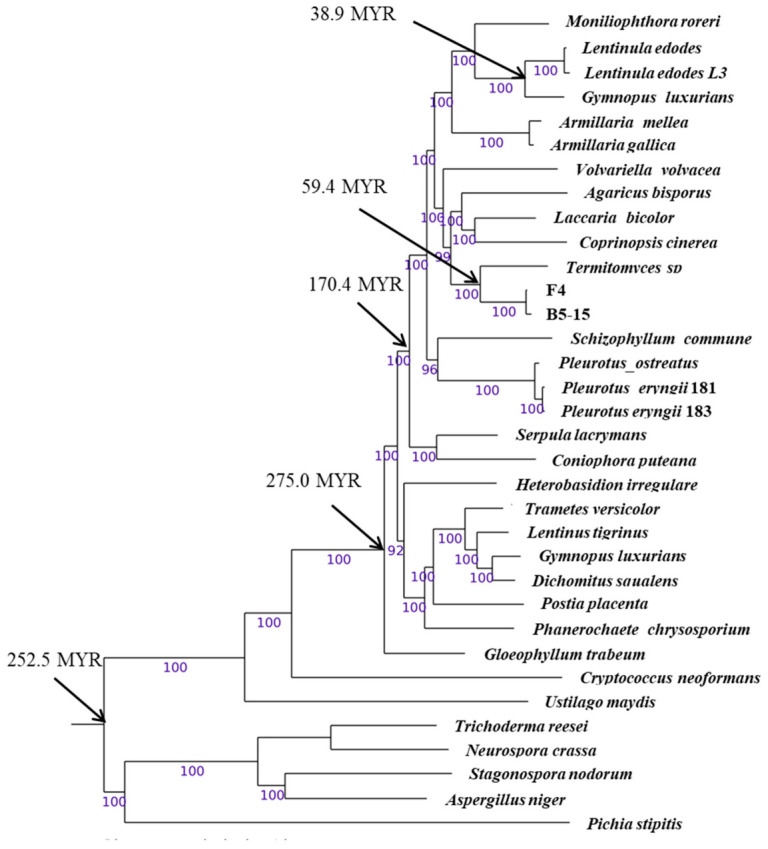
Phylogenetic and molecular dating tree of *H. marmoreus* and 32 other fungal species. Topology was inferred by Bayesian method based on concatenated single-copy orthologous proteins; node bootstrap values (1000 replicates) are shown on branches. Near-universal full bootstrap support results from rich informative sites of genome-scale orthologous supermatrix, while one node with BS = 92 reflects minor topological ambiguity. The arrows indicate the divergence time.

The genomes of the strains F4 and B5-15 were compared with 32 other reference genomes. A total of 35,723 gene families were clustered, of which 398 gene families were common in all the genomes. Subsequently, 107 single-copy orthologous genes were identified. After MAFFT alignment and trimAL filtering of conserved-site, the final supermatrix contained a large number of phylogenetically informative amino acid sites derived from dozens of single-copy genes. Such a large-scale genome-derived dataset provided abundant evolutionary signals to resolve phylogenetic relationships robustly. Consequently, most nodes gain full bootstrap support (BS = 100, 1000 replicates). Only one node exhibited a bootstrap value of 92. The results revealed that *H. marmoreus* clustered with other Agaricales species and had the closest evolutionary affinity with *Termitomyces* sp. (Figure 2). The divergence time between *H. marmoreus* and *Termitomyces* sp. was estimated at 59.4 MYA, with the split occurring around the Paleocene Epoch.

### 2.4. Plant Cell Wall Degradation

**Figure 3:**
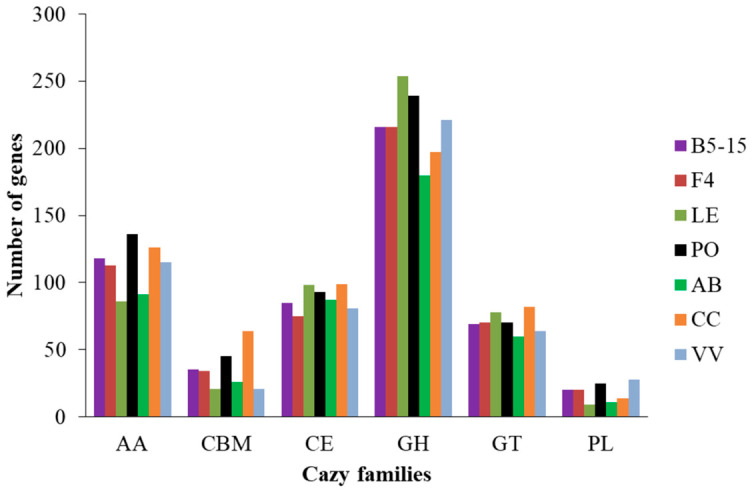
The number of 6 Cazy families in the 7 genomes. AA: Auxiliary activities, CBM: carbohydrate-binding modules, CE: carbohydrate esterases, GH: glycoside hydrolases GT: glycosyl transferases, PL: polysaccharide lyases. LE: *Lentinula edodes*; PO: *Pleurotus ostreatus*; AB: *Agaricus bisporus*; CC: *Coprinopsis cinerea*; VV: *Volvariella volvacea*.

The sets of CAZyme family genes related to plant cell wall degradation detected in the two genomes were similar (Figure 3). A total of 543 and 528 CAZyme-coding genes were identified in strains B5-15 and F4, respectively. Both strains contained the same number of glycoside hydrolase (GH) and polysaccharide lyase (PL) families. Only a one-gene difference was detected in carbohydrate-binding modules (CBMs, 35 copies in B5-15 and 34 copies in F4) and glycosyl transferases (GTs, 69 copies in B5-15 and 70 copies in F4). The most striking difference between the two strains was in carbohydrate esterases (CEs), with B5-15 harboring 10 more genes (85 copies) than F4 (75 copies). Additionally, the gene copy number of auxiliary activities (AAs) was also different, that is, 118 in B5-15 and 113 in F4.

Compared with other species (other whit-rot and straw-rot fungi), the total number of CAZymes varied widely, ranging from 455 copies in *A. bisporus* to 608 in *P. ostreatus*. The number of CAZyme families in *H. marmoreus* were less abundant than those in *L. edodes* and *P. ostreatus* but more abundant than in the other two straw-rot species, *A. bisporus* and *C. cinerea*. And the average number of genes belonging to GH families was much higher in white-rot fungi (*P. ostreatus*, *L. edodes* and *H. marmoreus*, Figure 3) than in straw-rot or litter-rot fungi (*A. bisporus*, *V. volvacea* and *C. cinerea*).

The genes encoding cellulases, hemicellulases, pectinases and lignin oxidases were listed in Table 2. A total of 18 and 17 candidate cellulase genes were identified in the genomes of the B5-15 and F4 strains, respectively, with the only one difference being in β-glucosidase (9 genes in B5-15 vs. 8 in F4). The most abundant multicopper oxidase genes were observed in *H. marmoreus* (20 copies), of which 19 were laccases. For lignin-degrading auxiliary enzyme, B5-15 contained 3 more genes (47 copies) than in F4 (44 copies), and the largest difference found in aryl-alcohol oxidase (35 in B5-15 and 31 in F4). The numbers of genes related to hemicellulose (20 copies), pectinase (14 copies) and lignin oxidase (23 copies) were identical in both strains.

There are differences in lignocellulolytic enzyme genes between wood-rotting fungi and straw-rotting fungi. In the white-rot fungi (*P. ostreatus*, *L, edodes* and *H. marmoreus*), the cellulase and pectinase genes were more abundant than in straw rot fungi (*A. bisporus*, *V. volvacea* and *C. cinerea*). In contrast, the three straw-rot fungi had more hemicellulase genes (average number 33) than the white-rot fungi (average number 18). The numbers of genes encoding lignin oxidase and lignin-degrading auxiliary enzymes were slightly higher in white-rot fungi than in the three straw-rot species. However, there were no genes encoding lignin peroxidases and pectin lyases in any of the genomes used in this study.

### 2.5. MatA and MatB Gene Loci

**Figure 4:**
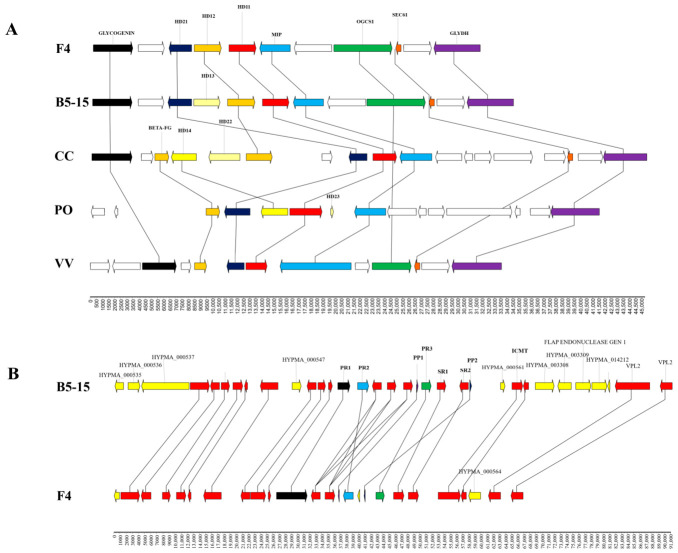
Structure comparisons of the A mating type locus and the B mating type locus in different genomes. (**A**) A mating type locus; homologous genes were connected with lines; arrows indicate the putative direction of transcription; red boxes indicate that genes were conserved among the three species; green boxes indicate that genes were conserved between two species; yellow boxes indicate that genes are conserved between two species. (**B**) B mating type loci. PP: pheromone precursor; PR: pheromone receptor. Genes of the same color connected by lines represented homologous genes.

The genetic diversity of the *MatA* and *MatB* gene loci was considerably higher than that of other species based on genomic data. The A-mating-type locus HD1 and HD2 homeodomain transcription factors were located in the same scaffold in both strains. The strain F4 possessed two HD1 domains and one HD2 domain. The HD2 domain was located 204 bp upstream of the HD1-2 domain, while the HD1-1 and HD1-2 domains were separated by a 600 bp region. In contrast, there were three HD1 domains and one HD2 domain in the genome of B5-15. The three HD1 domains were closely linked, separated by 625 bp and 620 bp regions, respectively. And the HD2 domain was located 189 bp upstream of the HD1-3 domain. The mitochondrial intermediate peptidase (*MIP*) genes were located downstream of the HD1-1 homeodomain, separated by distances of 307 bp and 363 bp in strains B5-15 and F4 respectively. The comparison of the genomic regions flanking A-mating-type locus in F4 and B5-15 with corresponding regions in other tetrapolar edible fungi (*P. ostreatus*, *C. cinerea*), and the bipolar fungus *V. volvacea*, revealed poor synteny (Figure 4A). There were two HD2 domains and 1 HD1 domain present in the genome of *P. ostreatus*, two HD2 domains and two HD1 domains in C. cinerea. Only one HD2 domain and one HD1 domain were found in V. volvacea (Figure 4A).

Using nr and SWISS-PROT annotations, five pheromone-receptor-like gene homologs *(PRs)* were identified in both genomes of *H. marmoreus*. Subsequent analysis of transmembrane structures, which filtered the proteins lacking seven transmembrane domains, resulted in the retention of three PRs per genome. The PRs located on scaffold 15 in strain B5-15 and on scaffold 46 in strain F4. Two pheromone precursor genes (*PPs*) were found in the same scaffold with PRs in each strain. The spanning physical distance of the *B* locus (PRs and PPs) was approximately 11,238 bp in F4 and 24,744 bp in B5-15 (Figure 4B). In strain B5-15, the physical distances between PR1 and PR2, PP1 and PR2, PP1 and PR3, PR3 and PP2 were 2069 bp, 8256 bp, 928 bp and 6111 bp, respectively. PR1 and PP1, PR3 and PP2 were separated by three hypothetical proteins and two STE-like proteins, respectively (Figure 4B). PR1 and PR2, PP1 and PR3 were closely linked. In strains F4, PP1 and PR2, PP2 and PR3 were closely linked (Figure 4B). The physical distances between PR1 and PP1, PP1 and PR2, PR2 and PP2, and PP2 and PR3 were 5076 bp, 656 bp, 1782 bp, and 1777 bp, respectively (Figure 4B). The synteny of the B locus between the two strains was notably poor. The open reading frames of both PPs and PR2 were oriented in opposite directions in the two strains, which might contribute to the considerably higher diversity of the B locus, suggesting that inversions of B locus-related genes may occur in *H. marmoreus*.

### 2.6. Melanin Biosynthesis and Related Gene Expression in the Brown Variety

**Figure 5:**
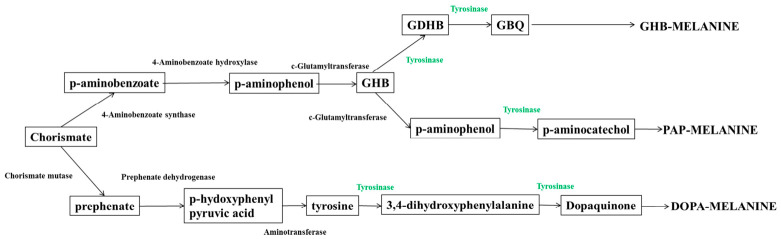
Genes presumed to be involved in the melanin biosynthesis pathways in B5-15. Tyrosinase labeled with green revealed that it was absent in the strain F4.

A search was undertaken to identify homologous genes involved in melanin biosynthesis in both genomes (Table 3). The results revealed that strain B5-15 could biosynthesize melanin using chorismate as the starting molecule via the p-aminobenzoate and prephenate branches (Figure 5). Chorismate could be converted into prephenate by chorismate mutase (1 predicted gene in the genome). The prephenate was converted into p-hydroxyphenyl pyruvic acid by prephenate dehydrogenase. Tyrosine subsequently was synthesized in order by phenylpyruvate aminotransferase using p-hydroxyphenyl pyruvic acid as the substrate. Tyrosinase was responsible for converting tyrosine to 3,4-dihydroxyphenylalanine and dopaquinone. In the second p-aminobenzoate branch, a total of one 4-aminobenzoate synthase gene, four 4-aminobenzoate hydroxylase genes, two c-glutaminyltransferase genes and one tyrosinase gene were identified. The 4-aminobenzoate hydroxylase was involved in the conversion of p-aminobenzoate into p-aminophenol. Then, p-aminophenol was catalyzed to GHB. GHB-melanin and PAP-melanin were then produced by tyrosinase and c-glutamine transferase using GHB as the substrate. In both branches, tyrosinase was the most important and carried out catalysis in several steps. However, in the white variety F4, genes related to tyrosinase were not found, the absence of which might lead to the failure of melanin biosynthesis in the white variety (Table 3).

KEGG annotations of the two genomes (B5-15 and F4) revealed that 15 and 13 genes were involved in tyrosine metabolism, respectively (Table S2). The genes absent in the white F4 strain, including alcohol dehydrogenase, primary-amine oxidase, homogentisate 1,2-dioxygenase, tyrosinase and phenylpyruvate tautomerase, were enriched in the B5-15 strain (Figure S1). The information obtained from Figures S1 (B5-15) and S2 (F4) revealed that tyrosinase was responsible for converting tyrosine into dopaquinone and L-DOPA and subsequently converted dopaquinone into indole-5,6-quinone. Tyrosinase played important roles in the process of melanin biosynthesis.

### 2.7. Expression of Genes Involved in Melanin Biosynthesis

The gene expression levels involved in melanin biosynthesis were also evaluated by RNA-Seq and RT-qPCR (Table 4 and Table 5). Tyrosinase, a key enzyme, showed the highest expression at the HP stage, with expression levels 2.4-fold and 13.70-fold higher than those at HM and HF, respectively. Chorismate mutase was expressed at higher levels in HF than in HP. The aminotransferase gene (scaffold 9.g291) exhibited peak expression at HP (FPKM = 36.17) and HF (FPKM = 32.69) stages compared with HM (FPKM = 11.95). The 4-aminobenzoate hydroxylase gene (scaffold1.g603) was more highly expressed at HM, while the 4-aminobenzoate synthase gene (scaffold 1.g494) showed higher expression at HF (FPKM = 60.96) than at HM (FPKM = 31.10) and HP (FPKM = 36.15). The c-Glutamyltransferase gene reached its highest expression at HF stage. qRT-PCR validation confirmed similar expression patterns of selected genes (Table 5).

### 2.8. LC/MS-Based Untargeted Metabolomics

A total of 3104 metabolites belonging to 345 superclasses were detected in both white and brown varieties (Table S3). Among these 300 amino acids, peptides, and analogs, 149 fatty acids and conjugates, 132 carbohydrates and carbohydrate conjugates, 66 flavonoids, 63 glycerophosphoserines and 49 fatty alcohols were identified (Table S4). Arachisprenol 10 and 11 were the most abundant in both varieties (Table S3), followed by PE (17:1(9Z)/20:0), solanesol and arachisprenol 12. The top seven metabolites were all from the class lipids and lipid-like molecules (Table S3).

**Figure 6:**
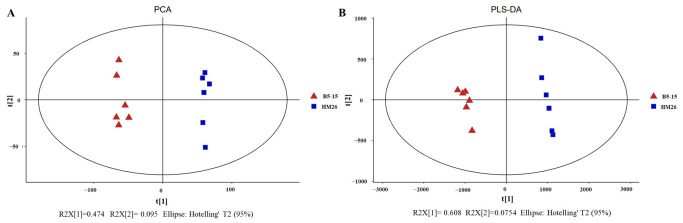
PCA and OPLS-DA analysis of metabolite contents in fruiting bodies of brown B5 and white HM26. (**A**) PCA; (**B**) OPLS-DA.

The metabolomic profiling showed markedly different metabolomes between the two strains based on PCA (R^2^X = 0.569, Figure 6A) and OPLS-DA analysis (R^2^X = 0.684, R^2^Y = 0.996 and Q^2^ = 0.982, Figure 6B). Using *t*-test statistics, 243 different metabolites belonging to nine known superclasses were identified between the two varieties, of which 119 were upregulated and 124 downregulated in the white variety (Tables S5 and S6). Organooxygen compounds, carboxylic acids and derivatives were the most abundant in white strain HM26 (Table S7). Meanwhile, fatty acyls and glycerolipids were higher in brown strain B5 (Table S8).

Among the 243 different metabolites, 20 amino acid metabolites were detected in HM26, 11 upregulated and nine downregulated (Table S9). The contents of six flavonoid metabolites were different in both strains. Isoliquiritigenin 2′-glucosyl-(1-4)-rhamnoside was considerably more abundant in HM26 than in B5 (Table S10). Meanwhile, the contents of 6-methoxykaempferol 3,7-bis(3-acetylrhamnoside) and luteolin 7-glucosyl-(1-6) –(4′-caffeoylglucoside) were higher in B5 than in HM26 (Table S11). Comparisons of terpenoid compounds revealed that one monoterpenoid, one diterpenoid, two sesquaterpenoids and two riterpenoids were different (Table S12). With the exception of S-furanopetatin and 6-geranylgeranyl 8′-methyl 6,8′-diapocarotene-6,8′-dioate, the contents of camelliol C, 4,4-dimethyl-5α-cholesta-8,24-dien-3β-ol, 1,1,2-trimethyl-3,5-bis(1-methylethenyl)cyclohexane, and phytoene 1,2-epoxide were considerably higher in HM26 than in B5 (Table S12). The KEGG enrichment revealed that these different metabolites were involved in 43 pathways, including tyrosine metabolism. In tyrosine metabolism, melanin biosynthesis was also the most notable branch in brown strains (Table S11).

Pearson correlation analysis with the top 50 individual metabolite−metabolite correlations between the two varieties revealed 20 positive and 30 negative correlations (Figure S4, Table S13). Lipids and lipid-like molecules were the most abundant in these metabolites followed by organic acids and derivatives and organic oxygen compounds (Table S14). Sterol lipids, steroids and steroid derivatives and sphingolipids were the most numerous in the significant positive correlation group (Table S13). Glycerolipids, prenol lipids and glycerophospholipids predominated in the significant negative correlation group (Table S13).

## 3. Discussion

In this study, genomic comparison between strains F4 and B5-15 revealed minor differences in CAZyme families and lignocellulose-degrading enzymes. A similar result was obtained from the comparisons of lignocellulose-degrading enzymes in *L. edodes*, where also only minor differences were observed between the two compatible strains 135A and 135B (compatible monokaryotic strains isolated from the dikaryotic strain 135) [20]. However, the dependence of compatibility on relationships of degradation ability between the strains needs to be further studied. A large difference in rDNA copy number between strains was detected, as commonly observed in other fungi, i.g. *L. edodes* [44], *A. bisporus* [45] and *Strobilomyces alpinus* [46]. It is reported that 27% of macrobasidiocarp-forming mushrooms harbor polymorphic rDNA in their genomes, and 65% of these wild species exhibit substantial intraspecific disparities in rDNA copy number [47]. Internal causes for fungal rDNA copy divergence include incomplete concerted evolution, unequal crossover and transposon insertion; external factors involve habitat stress and geographic isolation screening. Artificial breeding and successive asexual propagation further aggravate intraspecific copy number variation in cultivated strains [47,48].

A high number of multicopper oxidase genes (laccase) were found in *H. marmoreus* (20 copies) [15], a feature traditionally considered characteristic of lignin-degrading white-rot fungi [49]. The result is in agreement with previous studies confirming *H. marmoreus* as a wood-decay fungus [12,13,14]. However, no lignin peroxidases were found in any of the strains, which is also consistent with other studies based on genomic analysis and PCR amplifications [31,50,51]. More lignin oxidases and degrading auxiliary enzymes participating in lignin decomposition were identified in white rot fungi in this study, which might reflect their high abilities to degrade lignin [17,52]. In addition, there were also a large number of laccase genes in straw-rot or litter-rot fungi, particularly in *C. cinerea* and *V. volvacea*, and the copy number was higher than that of two white-rot fungi, *P. ostreatus* and *L. edodes* as reported in a previous study [31]. The wood decay mechanisms seem to be considerably more diverse than initially believed, and the diagnostic separation of rot mechanisms in fungi into white rot and straw rot might need to be revisited [53].

In a tetrapolar heterothallic fungus, the loci matA and matB regulate the heterokaryotic fusion [23,54]. In *H. marmoreus*, poor synteny of the putative mating-type loci and their flanking regions compared with the three other fungi used in this study revealed that varied mating types are present in edible mushrooms. The most striking finding was that the number of A mating loci (with HD domains varying from two to five) differed between the two *H. marmoreus* varieties, which is consistent with previous studies and with results obtained in other mushrooms [25,31,33,35]. Many studies have revealed that the diversity of the mating type B locus in mushrooms is considerably higher than that of the A locus [33]. The rearrangement of the B locus might occur in *H. marmoreus*. The varied numbers and rearrangement of mating-type loci may have originated from the differentiation of the *H. marmoreus* strains [35]. The high diversity of the A and B loci ensured the crossbreeding probability in the wild environments. Understanding the mating-type loci may help us to uncover the reproductive mode of *H. marmoreus* and facilitate breeding efforts [23].

The metabolites in the fruiting bodies of *H. marmoreus* were abundant, with most kinds of metabolites belonging to the class amino acids, peptides, and analogs as revealed by other studies [7,55,56,57,58,59,60]. In a GC-TOF-MS study, 20 out of 43 metabolites were amino acids [8]. Amino acids are highly associated with taste in mushrooms, e.g., bitter, sweet, and monosodium glutamate-like taste [61,62]. The diversity of amino acids, peptides, and analogs may be responsible for the taste of *H. marmoreus* [8,63]. The other abundant metabolites were lipids and lipid-like molecules in this mushroom, which may play important roles in membrane biosynthesis, including pileus expansion and fungal chitin wall synthesis [59,64]. The white and brown varieties showed different metabolite profiles based on metabolomics, which was also consistent with previously published research [65,66]. The statistics between the two varieties suggested that organooxygen compounds, carboxylic acids and derivatives were considerably higher in the white strain, while fatty acyls and glycerolipids were higher in the brown strain. In addition, the contents of some other flavonoid and terpenoid metabolites differed between the two varieties. Variations in these metabolites may account for the observed differences in flavor.

BLAST results, along with genomic and metabolomic KEGG enrichment analyses, revealed that tyrosine metabolism played a key role in melanin biosynthesis in the brown variety, with tyrosinase serving as the essential enzyme. Tyrosinase is widely involved in the L-DOPA pathway in fungi [67], including mushrooms, e.g., *L. edodes* [68], *G. frondosa* [43] and *A. bisporus* [69]. Owing to the absence of tyrosinase in the white variety, melanin production failed. In brown *H. marmoreus*, tyrosinase expression was highest at the primordium stage and lowest at the fruiting body stage in this study, which was consistent with the color transition period [70]. Another study also revealed that tyrosinase exhibited the same expression patterns. In *G. frondosa*, the expression level of tyrosinase increased rapidly in the stage of primordia and peaked in young fruiting bodies (coloration stage), then decreased dramatically in mature fruiting bodies [43]. In another study, a cytochrome P450 was selected as the candidate causal gene for the melanogenesis for L-DOPA pathway, suggesting that melanogenesis may be a quantitative trait and a complex biological process [40]. All the results suggested that melanin formation before fruiting body maturity accounts for the brown or gray color. Besides pigmentation, melanin has many other biological functions, including associations with the cell wall and cytoplasm, to protect the fungus from environmental insults [71,72]. However, melanogenesis in *H. marmoreus* requires further investigation.

## 4. Materials and Methods

### 4.1. Monokaryotic Strain Isolation, Culture Conditions and Industrialized Cultivation

Two monokaryotic strains, F4 and B5-15 deposited at the Institute of Edible Fungi, Shanghai Academy of Agricultural Sciences, were isolated from widely cultivated dikaryotic strains, the white and brown strains HM26 and B5, respectively, using the protoplast isolation method [73]. When B5-15 was crossed with any monokaryon, the resulting strain was brown; while F4 produced white fruiting bodies only when crossed with white-type monokaryons. Therefore, B5-15 and F4 represented the brown and white varieties, respectively. The fungi were maintained on potato dextrose agar (PDA) at 25 °C.

Dikaryotic strains HM26 and B5 were cultivated for artificial cultivation according to previously published methods [39]. The substrate for both strains consisted of Douglas fir sawdust, corncobs, soybean hulls, and wheat bran at a 40:30:15:15 ratio (*v*/*v*). Samples were grown for 80 days at 20 ± 1 °C, with samples being moved to an LED growth chamber after primordia formed. The fruiting bodies were harvested on day 120.

### 4.2. DNA Sequencing, Data Processing, Genome Assembly and Annotation

The total genomic DNA of a 10-day-old PDA culture was extracted using the DNeasy Plant Mini Kit (Qiagen, Courtaboeuf, France). To improve the genome assembly, two separate platforms, Illumina (San Diego, CA, USA) and PacBio (Pacific Biosciences, Menlo Park, CA, USA), were used. All sequencing was conducted by Personalbio Company (Shanghai, China). Sequence library building and raw data trimming were performed according to previously published methods [74]. Both of the filtered datasets were assembled into scaffolds using SPAdes-3.5.0, with k-mer sizes of 21, 33, 55, 77, 99, 111, and 127, and the careful option was enabled to reduce misassemblies [75]. The method used for gene model prediction was based on Augustus [76]. Annotation of predicted genes was performed using BLAST (Basic Local Alignment Search Tool) searches against NCBI’s nonredundant protein database (“nr”). Gene Ontology and KEGG metabolic pathway matches were carried out using local Blast2go tools [77] and KAAS [78], respectively. All predicted protein families were analyzed by InterproScan [79] and Pfam analysis [80]. Carbohydrate-active enzymes were annotated using dbCAN [81]. Oxidoreductases were extracted from the proteins predicted from the genome using a combination of IPR domain searches and the JGI cluster pipeline (http://genome.jgi.doe.gov/31_SAP, accessed on 3 March 2021) according to the method published previously [17]. These genome sequences have been deposited at the NCBI (http://www.ncbi.nlm.nih.gov/, accessed on 6 May 2020) under the accession numbers SSXQ00000000 and SSXR00000000.

### 4.3. RNA Sequencing and Data Processing

To investigate gene expression patterns of B5 strain across developmental stages, RNA sequencing was performed on samples collected from three growth phases: mycelium (HM, cultured for 67 days), primordia (HP, cultured for 75 days), and fruiting bodies (HF, cultured for 79 days). Total RNA from 12 samples (3 replicates per stage) was extracted using TRIzol Reagent (Invitrogen, Carlsbad, CA, USA). mRNA purifications, fragmentations, cDNA synthesis, library preparations and transcriptome sequencing were performed at Personalbio Company (Shanghai, China).

The 3′ adaptor sequences of paired-end raw reads generated from the Illumina platform were removed using Cutadapt 1.9.1 [82]. Low-quality reads with quality scores < 20 and duplicate reads caused by PCR amplifications were filtered for later analysis. The high-quality reads were mapped to the reference genome B5-15 via the software Hisat2 2.0.1 [83]. The level of gene expression was measured in terms of fragments per kilobase of transcript per million mapped reads (FPKM). Differentially expressed genes with >2-fold change (FC) and >1 fragment per kilobase of transcript million (FPKM) were selected for further KOG and KEGG enrichment analyses.

### 4.4. Identification Genes of Mating and Melanin Biosynthesis

The A-mating-type genes were identified using previously published methods [31]. The protein dataset was blasted against the matA and MIP genes of *L. edodes* and *Schizophyllum commune*. The pheromone receptor genes were also determined using blast searching of the Swiss-Prot and nr databases. The short-sequence pheromone precursors were annotated based on the Pfam domains. The synteny of the mating-type gene locus was examined using ChromoMapper (http://www2.unil.ch/biomapper/chromomapper/, accessed on 4 July 2024).

The genes for melanin biosynthesis were detected using BLAST against a self-built database using a previously published method [36]. The transcript levels of target genes were determined by RT-qPCR using a QuantStudio™ 5 System (Thermo Fisher Scientific, Waltham, MA, USA). The actin gene of *H. marmoreus* was selected as the internal reference gene, and RT-qPCR primers were designed via Primer3Plus (Table 6). The qPCR assay was carried out using SuperStar Universal SYBR Master Mix (CWBIO, Beijing, China). The 20 μL PCR reaction system consisted of 10 μL of 2× SuperStar Universal SYBR Master Mix, 0.4 μL of forward primer (10 μM), 0.4 μL of reverse primer (10 μM), 1 μL of cDNA template, and 8.2 μL of ddH_2_O. The thermal cycling program was set as follows: initial denaturation at 95 °C for 30 s, followed by 40 cycles of denaturation at 95 °C for 15 s and annealing at 60 °C for 30 s. The relative gene expression levels were quantified using the 2^−ΔΔCt^ method.

### 4.5. Phylogenomic Analysis

For phylogenetic analysis, a total of 34 fungal species belonging to Basidiomycota and Ascomycota were used (including F4 and B5-15). Protein sequences from these species were compared using BLASTP with an e-value cutoff of 1 × 10^−5^ and a maximum of 500 hits per query. The BLASTP results were then processed with OrthoMCL v5 [84] under default parameters to identify orthologous gene families.

Multiple sequence alignments of these single-copy orthologous genes were performed using MAFFT, and the aligned sequences from each species were concatenated into a single long sequence. Conserved blocks within the concatenated alignment were extracted using trimAL [85] with default parameters. A phylogenetic tree was constructed from this conserved alignment using MrBayes v3.2.1 with a mixed amino acid model, gamma-distributed rate variation across sites and a proportion of invariable sites [86].

For molecular clock analysis, three fossil calibration points were applied [77]: the most recent common ancestor (MRCA) of *Coprinopsis cinerea*, *Laccaria bicolor*, and *Schizophyllum commune* was set at 122.74 million years ago (MYA); the MRCA of *Serpula lacrymans* and *Coniophora puteana* was set at 104.23 MYA; and the MRCA of *Pichia stipitis*, *Aspergillus niger*, *Cryphonectria parasitica*, *Stagonospora nodorum*, and *Trichoderma reesei* was set at 517.55 MYA [17,31]. Divergence times for other nodes were calculated using r8s v1.80 with the TN algorithm (truncated Newton), the penalized likelihood (PL) method, and a smoothing parameter of 1.8 determined by cross-validation [87].

### 4.6. Metabolic Sample Preparation and LC-MS Analysis

A total of 12 mature fruiting body samples were collected from white HM26 (6 samples) and brown B5 (6 samples) varieties. Sample preparations and LC-MS analysis were conducted according to the methods published previously [88]. Metabolites were identified using progenesis QI based on public Lipidmaps and self-built databases. Principal component analysis (PCA) and orthogonal partial least-squares-discriminant analysis (OPLS-DA) were applied to visualize the relationship between white and brown varieties using R packages. Hotelling’s T2 region, shown as an ellipse in the score plots of the models, defined the 95% confidence interval of the modeled variation. The R2X or R2Y and Q2 values were used to describe the quality of the models. A default seven-round cross-validation in SIMCA was performed throughout to determine the optimal number of principal components and to avoid model overfitting. The OPLS-DA models were also validated by a permutation analysis (200 times). The different metabolites were selected based on the variable influence on projection (VIP) values and *p* values obtained from the OPLS-DA model with a two-tailed Student’s *t*-test on the normalized peak areas. Metabolites with VIP values greater than 1.5 and FDR-adjusted *p* values less than 0.05 were defined as significantly different.

## 5. Conclusions

The genomic and metabolomic information of the different *H. marmoreus* varieties (brown and white) revealed that some backgrounds of the two varieties were different, e.g., rDNA, mating type genes and metabolites. Information obtained from this study may help to elucidate the divergence between the two varieties and may facilitate breeding efforts.

## Figures and Tables

**Table 1:** Genome assembly statistics for *H. marmoreus* derived from Illumina and PacBio data.

Feature	F4	B5-15
Genome size (bp)	40,851,948	41,902,673
Scaffold Num.	98	92
Scaffold N50	819,258	1,234,483
GC content (%)	49.45	49.64
Predicted genes	12,411	12,618
tRNA genes	331	301
5S rDNA genes	3	88
5.8S rDNA genes	4	93
18S rDNA genes	2	94
28S rDNA genes	4	97
BUSCO	95.86%	96.93%

**Table 2:** CAZyme families involved in the degradation of cellulose, hemicellulose and pectin in the 7 fungal genomes.

Class	Enzyme	EC	CAZyme Families	B5-15	F4	LE	PO	CC	VV	AB
Cellulase	Endo-β-1,4-glucanase	3.2.1.4	GH12, GH45, GH5_5, GH9, AA1_1, GH3, GT35, GH44	7	7	5	9	4	4	4
	1,4-β-Cellobiosidase	3.2.1.91	GH6,GH7	1	1	0	3	5	5	1
	β-Glucosidase	3.2.1.21	GH1,GH3, AA3_3, GH11, GH16, GH18	10	9	11	12	9	13	5
	Total			18	17	16	24	18	22	10
Hemicellulase	Endo-1,4-betaxylanase	3.2.1.8	GH3,GH10,GH11	8	8	0	5	12	18	4
	β-Xylosidase	3.2.1.37	GH3,GH43, GH5	4	4	5	5	4	3	5
	α-Glucuronidase	3.2.1.131	GH67	1	1	1	1	1	3	2
	Acetylxylan esterase	3.1.1.72	CE1,CE3,CE4, CE5,CE12	5	5	1	7	18	10	7
	Feruloyl esterase	3.1.1.73	CE1	0	0	0	0	0	0	0
	α-Larabinofuranosidases	3.2.1.55	GH43, GH51,GH62	2	2	3	5	4	6	2
	Total			20	20	10	23	39	40	20
Pectinase	Pectin lyase	4.2.2.10	PL1	0	0	0	0	0	0	0
	Pectate lyase	4.2.2.2	PL1, PL3	9	9	3	11	3	13	3
	Pectinesterase	3.1.1.11	CE8	3	3	3	2	0	3	2
	Polygalacturonase	3.2.1.15	GH28	2	2	5	1	0	0	1
	Total			14	14	11	14	3	16	6
Lignin Oxidase	Multicopper oxidase	1.10.3.2	AA1	20	20	13	12	17	11	9
	Lignin peroxidase	1.11.1.14	AA2	0	0	0	0	0	0	0
	Manganese peroxidase	1.11.1.13	AA2	2	2	8	5	1	2	2
	Other peroxidase	1.11.1.16	AA2	1	1	1	5	2	8	3
	Total			23	23	22	22	20	21	14
Lignin degrading Auxiliary enzyme	Aryl-alcohol oxidase	1.1.3.7	AA3_2a	35	31	13	35	21	25	16
	Glucose oxidase	1.1.3.4	AA3_2b	4	5	2	0	2	0	0
	Alcohol oxidase	1.1.3.13	AA3_3	2	2	4	4	2	5	4
	Pyranose oxidase	1.1.3.10	AA3_4	0	0	1	0	0	0	0
	Vanillyl-alcohol oxidase	1.1.3.38	AA4	0	0	2	1	0	1	1
	Glyoxal oxidase	1.1.3.-	AA5_1	5	5	4	12	5	3	0
	Galactose oxidase	1.1.3.9	AA5_2	0	0	0	0	0	0	0
	Benzoquinone reductase	1.6.5.6	AA6	1	1	2	2	3	2	4
	Total			47	44	28	54	33	36	25

Note: AB: *Agaricus bisporus*; VV: *Volvariella volvacea*; CC: *Coprinopsis cinerea*; PO: *Pleurotus ostreatus*; LE: *Lentinula edodes*.

**Table 3:** Number of identified genes of the two genomes and *Agaricus bisporus* genome for melanin biosynthesis.

Enzymes	AS	B5-15	F4
Chorismate mutase	1	1	1
4-Aminobenzoate synthase	2	1	1
4-Aminobenzoate hydroxylase	5	4	4
c-Glutaminyltransferase	2	2	1
Prephenate dehydratase	1	0	0
Prephenate dehydrogenase	1	1	1
(4-Hydroxy) phenylpyruvate aminotransferase	3	3	1
Tyrosinase	6	1	0
Phenylalanine ammonialyase	2	1	2
Trans-Cinnamate-4-monooxygenase	2	1	2
4-Coumarate CoA ligase	10	6	6
Catalase	3	2	2
Chloroperoxidase	1	0	0
Manganese peroxidase	1	2	2
Photoregulator B	1	1	1

Note: AS: *Agaricus bisporus*.

**Table 4:** The different gene expression of melanin biosynthesis in the brown variety.

Num.	Enzymes	Gene ID	HM	HP	HF
1	Chorismate mutase	scaffold6.g205	46.24 b	29.92 b	61.33 a
2	Aminotransferase	scaffold9.g291	15.77 b	36.17 a	32.69 a
3	Tyrosinase	scaffold5.g45	232.20 b	563.80 a	41.18 c
4	4-Aminobenzoate synthase	scaffold1.g494	31.10 b	36.15 b	60.96 a
5	4-Aminobenzoate hydroxylase	scaffold1.g603	42.35 a	2.50 b	5.01 b
6	c-Glutamyltransferase	scaffold13.g201	35.38 b	22.98 b	87.79 a

Note: HM: samples collected from the mycelium stage; HP: samples collected from the primordium stage; HF: samples collected from the fruiting body stage. Lowercase letters a, b and c revealed that they were different.

**Table 5:** RT-qPCR validation of related genes involved in melanin biosynthesis.

Num.	Enzymes	Gene ID	HM	HP	HF
1	Chorismate mutase	scaffold6.g205	1.00 ± 0.18 b	0.44 ± 0.05 c	1.39 ± 0.13 a
2	Aminotransferase	scaffold9.g291	1.00 ± 0.28 b	3.18 ± 0.75 b	13.54 ± 1.62 a
3	Tyrosinase	scaffold5.g45	1.00 ± 0.18 b	1.63 ± 0.16 a	0.41 ± 0.10 c
4	4-Aminobenzoate synthase	scaffold1.g494	1.00 ± 0.17 c	2.28 ± 0.25 b	4.04 ± 0.68 a
5	4-Aminobenzoate hydroxylase	scaffold1.g603	1.00 ± 0.11 a	0.65 ± 0.16 b	0.90 ± 0.13 ab
6	c-Glutamyl transferase	scaffold13.g201	1.00 ± 0.19 b	1.28 ± 0.15 b	2.30 ± 0.50 a

Note: HM: samples collected from the mycelium stage; HP: samples collected from the primordium stage; HF: samples collected from the fruiting body stage. Lowercase letters a, b and c revealed that they were different.

**Table 6:** The primers used for RT-qPCR.

Number	Genes	Sequences (5′-3′)
1	*scaffold6.g205*	F: ACCAGGCTGGAGGATACCAT
		R: GGGACTGGTATATCGCCGTG
2	*scaffold9.g291*	F: CCTACGGTTGCAATCCCACT
		R: GGGTAAAGGACCTGTTGCGA
3	*scaffold5.g45*	F: GCGTGCCAATCACAGAATCC
		R: CCAGATACGGTCGAGGTTCG
4	*scaffold1.g494*	F: GAACACCTGTTCGCTGGAGA
		R: GCTGAATGAGGAGCAGGGTT
5	*scaffold1.g603*	F: ACGCGCAAATATGGTCTTGC
		R: ATAGTGCCAAACTGGCTCCC
6	*scaffold13.g201*	F: CCCCGAGACTGGCATCATAC
		R: TAGTTGGTCGTGCACCCTTC
7	*Actin*	F: CCGAGCGGAAGTACTCTGTG
		R: ATGCTATCTTGCCTCCAGCC

## Data Availability

These genome sequences have been deposited at the NCBI (http://www.ncbi.nlm.nih.gov/, accessed on 3 March 2021) under the accession numbers SSXQ00000000 and SSXR00000000. And both the genomes have been submitted to the submission system as Supplementary Files. Raw resequencing data of RNA-seqs is deposited in the NCBI SRA (http://www.ncbi.nlm.nih.gov/sra, accessed on 6 May 2020) under accession number SRR11712018-SRR11712026. All the supplemental materials have been uploaded in GSA Figshare.

## Supplementary Materials

The following supporting information can be downloaded at: https://www.mdpi.com/article/10.3390/ijms27125372/s1.
