# Supplementary material for: Genomic and Metabolomic Comparisons Provide New Insights into Plant Cell Wall Degradation, Mating Diversity and Secondary Metabolites in Brown and White Commercial Hypsizygus marmoreus Varieties
Source: Int J Mol Sci. 2026 Jun 14;27(12):5372. doi: 10.3390/ijms27125372 (PMC13300218; doi:10.3390/ijms27125372)
Supplement: Supplementary file 1 [file ijms-27-05372-s001.zip › ijms-4261620-Supplement material.pdf]

International Journal of *Molecular Sciences*

MDPI

Supplementary Material

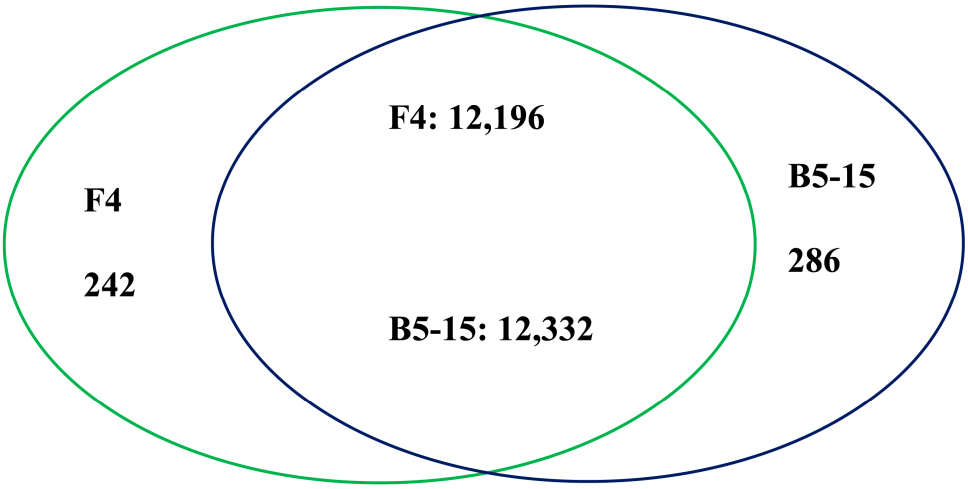

Figure S1. Venn diagram based on the predicted in the two stains F4 and B5-15.

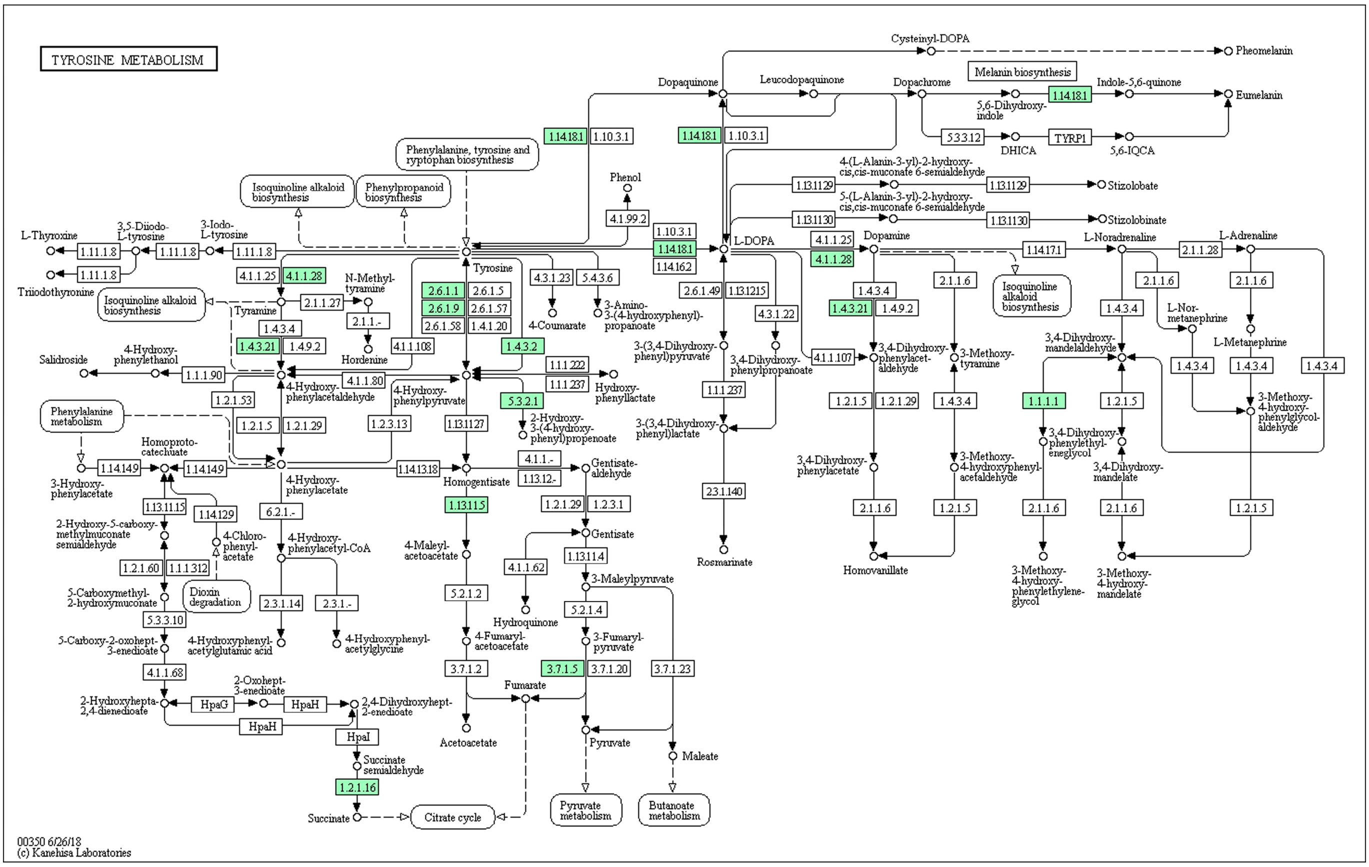

Figure S2. Genes involved in tyrosine metabolism enriched in KEGG annotation in strain B5-15. The nodes labeled green revealed the enriched genes or proteins.

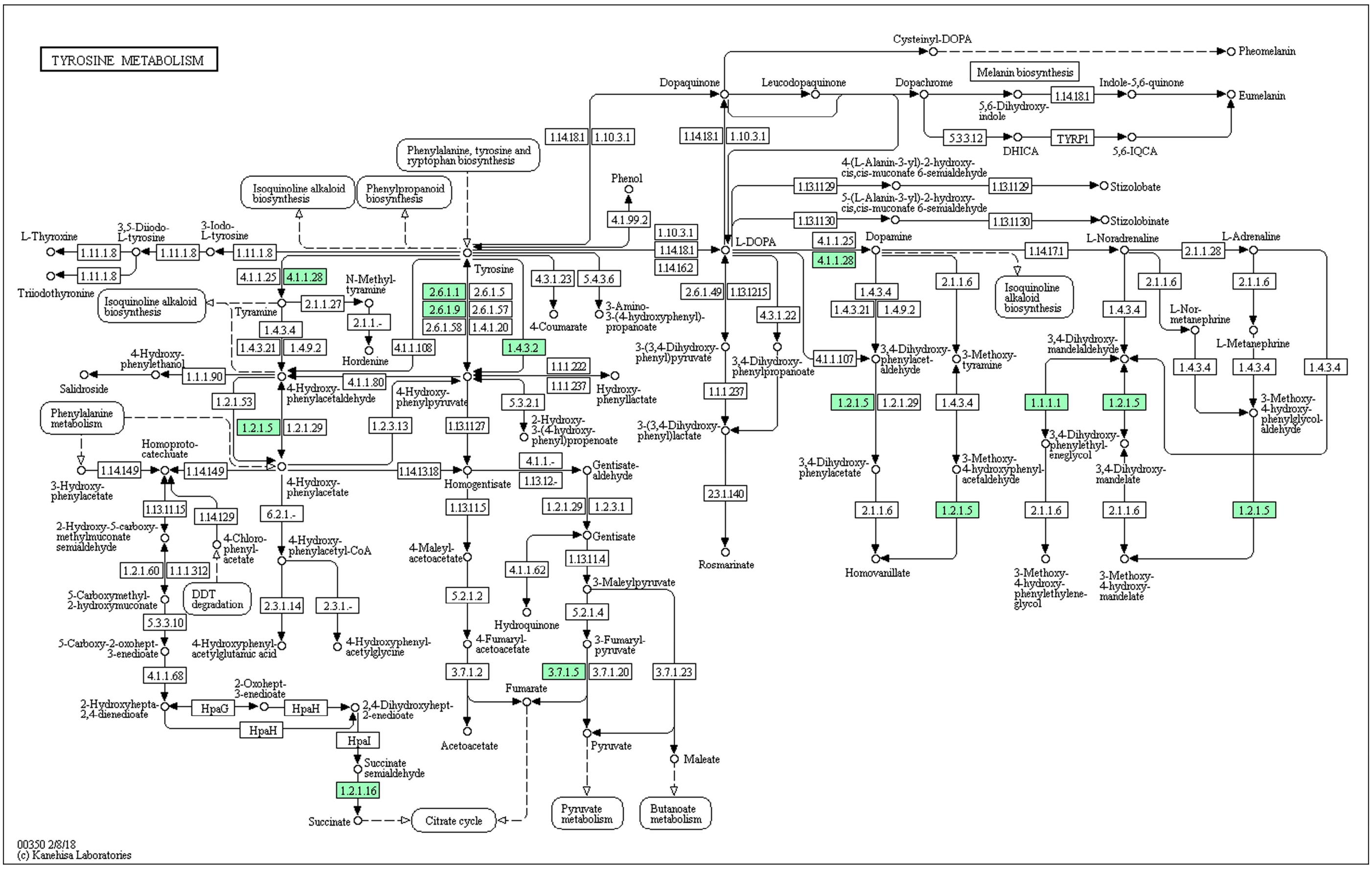

Figure S3. Genes involved in tyrosine metabolism enriched in KEGG annotation in strain F4. The nodes labeled green revealed the enriched genes or proteins.

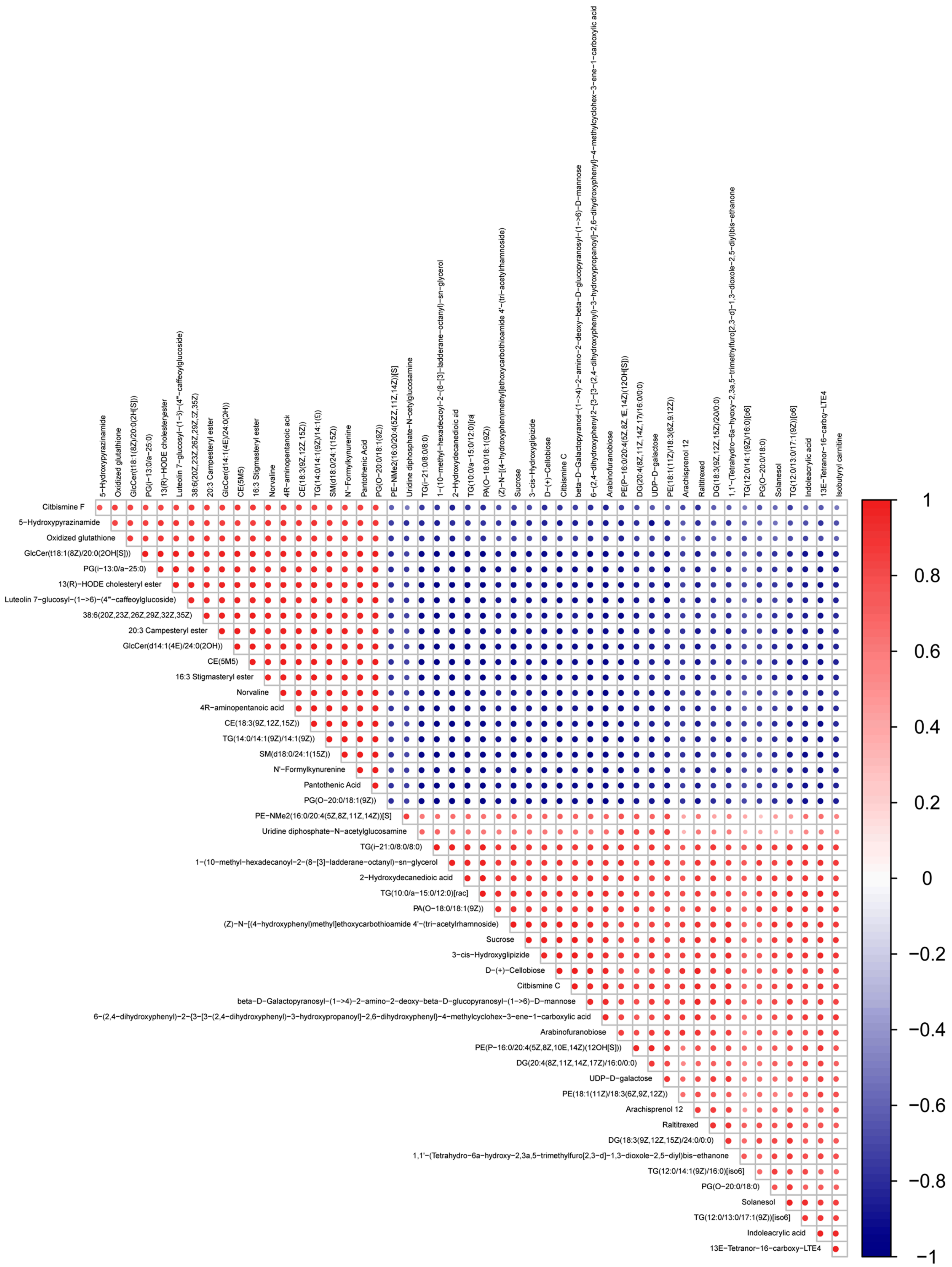

Figure S4. Pearson correlation analysis with the top 50 individual metabolite–metabolite correlations between the two varieties.
